# Novel Dietary Approaches for Controlling High Blood Pressure

**DOI:** 10.3390/nu12123902

**Published:** 2020-12-21

**Authors:** George Moschonis, Kalliopi Karatzi

**Affiliations:** 1 Department of Dietetics, Nutrition and Sport, School of Allied Health, Human Services and Sport, La Trobe University, Bundoora, VIC 3086, Australia; 2 Department of Nutrition and Dietetics, School of Health Science and Education, Harokopio University, 17671 Athens, Greece; pkaratzi@hua.gr

Hypertension is a common health problem, and one of the most important risk factors for cardiovascular disease. Hypertension treatment is usually based on drug administration, yet lifestyle changes and especially diet have also been proven to be almost as effective in hypertension therapy. Dietary components may act favourably in many pathophysiological mechanisms responsible for blood pressure elevation, both acutely and after long-term use. The Special Issue “Nutritional Therapy for High Blood Pressure” aimed at offering a valuable insight into how diet as a whole or via individual components can serve as valuable means for blood pressure control in patients or apparently healthy populations, thus bringing together some of the latest relevant scientific evidence.

The association of diet with blood pressure has been thoroughly investigated both holistically, through the examination of the whole diet and dietary patterns, but also at a single food item or nutrient level, via the examination of specific foods, nutrients and certain bioactive compounds. There is also variability with regards to the design of the studies that have provided evidence related to the association or the effects of nutrition on high blood pressure, considering that the Special Issue combines data from reviews, observational studies and clinical trials.

According to evidence coming from observational studies that examine the associations between the whole diet and blood pressure, the cross-sectional study by Valle et al. used data from the ESTEBAN survey that was conducted with a representative sample of French adults and reported that adhesion to the DASH (Dietary Approach to Stop Hypertension) diet was associated with lower systolic and/or diastolic blood pressure levels [1]. The DASH diet is proven for its effectiveness in lowering blood pressure, mainly due to the combination of many of the known blood pressure-reducing dietary factors in one dietary pattern, such as low sodium, high potassium, high fiber, low red meat and low dietary fat intake. Furthermore, this cross-sectional study also showed that people with the lowest adherence to the DASH dietary pattern, were associated with 1.8 mmHg higher systolic blood pressure (SBP) and 0.6 mmHg higher diastolic blood pressure (DBP) compared to those being on the highest quartile. Interestingly, some other studies have used lifestyle instead of dietary patterns, thus attempting to investigate an even more holistic approach regarding non- pharmacological means for hypertension prevention, such as the combination of healthy weight status, healthy diet, regular physical activity and alcohol consumption reduction. In this context, in the study by Lelong et al., 80,426 French adults participating to the NutriNet-Santé cohort study were examined prospectively to assess the incidence of hypertension after 3.5 years of follow-up [2]. This study used the Healthy Lifestyle Index to assess adherence to a healthy lifestyle and showed that the risk of hypertension decreases for each additional healthy lifestyle factor adopted by the participants. Furthermore, it was shown that adherence to all healthy lifestyle factors (i.e., healthy weight status, healthy diet, regular physical activity and reduction of alcohol consumption) decreased the risk of hypertension in half.

Other observational studies have reported associations between the dietary intake of specific nutrients and vascular outcomes related to high blood pressure. In this regard, the review by Tsirimiagkou et al. investigated the association between dietary sodium intake and major vascular damages, which include atheromatosis, arteriosclerosis and arterial remodeling, and lead among others also to hypertension [3]. Arteriosclerosis was much more investigated compared to atheromatosis and arterial remodeling, yet there were neither strong nor consistent results for any of those clinical conditions. Most importantly, the review by Tsirimiagkou et al. highlighted the methodological limitations mainly related to the inaccurate assessment of dietary sodium intake, as well as the absence of salt-sensitivity assessment, since salt sensitive and salt resistant study participants have been reported to have different or even inverse between sodium intake and hypertension. Therefore, serious concerns on the validity of the findings reported by these observational studies have been posed. Regarding alcohol intake it is well known that high alcohol consumption has an almost linear association with the risk of developing hypertension. The ESTEBAN survey showed that the positive association between alcohol consumption and blood pressure levels was only observed in men, while no such association was observed in women [1]. This finding paves the way for future research in this area, since it highlights the need to shed some more light on the sex differences indicated by the ESTEBAN survey, as well as by many other epidemiological studies that also indicate a different level of optimum alcohol consumption between men and women.

Considering the inappropriateness of observational studies to provide a causal pathway in the associations observed between the whole diet or its individual components and high blood pressure, clinical trials can more accurately address the therapeutic effect of diet, foods or single nutrients on hypertension. In terms of the blood pressure-lowering properties of certain food items, Cashman et al. conducted a randomized crossover pragmatic intervention trial on 97 apparently healthy adults in Ireland, with slightly to moderately elevated blood pressure [4]. These adults consumed either bread with “low-salt” (0.3 g salt/100 g) or bread with the typical level of salt (1.2%) as part of a 5-week cross-over design. Participants in the “low-salt” treatment decreased their salt intake by 1.7 g/day, while their SBP was significantly lower (by 3.3 mmHg) during the reduced-salt dietary period compared to the period when they were consuming bread with the typical level of salt. However, there were no differences observed in DBP. Similar findings were reported by another double-blind, randomized, controlled cross-over trial, which examined the effect of high polyphenol extra virgin olive oil on blood pressure and arterial stiffness in healthy Australian adults (OLIVAUS study). In this cross-over trial by Sarapis et al., participants were randomized to consume a daily dose of 60 mL of either high polyphenol olive oil (360 mg/kg polyphenols) or low polyphenol olive oil (86 mg/kg polyphenols) for three weeks. This study showed a significant decrease in peripheral and central SBP by 2.5 and 2.7 mmHg respectively, only after consumption of high polyphenol extra virgin olive oil. However, no within-group changes or between-group differences were observed in DBP and arterial stiffness markers. The use of refined olive oil instead of another type of vegetable oil in the control group probably explains the non-significant between-group differences in the OLIVAUS study. Although the concentration of polyphenols in refined olive oil is lower compared to extra virgin olive oil, it is still high enough to produce some clinically significant health benefits, also in terms of blood pressure and arterial stiffness. Following another approach, Jones et al. examined the effect of beetroot juice consumption on blood pressure, as well as on microvascular and macrovascular endothelial function markers in a randomized, double-blind, placebo-controlled pilot study in healthy older adults [5]. This pilot study showed that plasma nitrate (i.e., a strong vasodilator) increased significantly following two weeks of beetroot juice consumption, while SBP and DBP decreased by approximately 6 and 4 mmHg respectively compared to the placebo group. However, there were no other differences with regards to the effect of beetroot juice consumption on endothelium function markers.

The clinical trials published in this Special Issue, provide strong evidence that support the therapeutic potential of reduced salt (or sodium) intake through staple foods, as well as of bioactive nutrients, such as polyphenols in olive oil and nitrate in beetroot juice, in the management of hypertension. Nevertheless, the blood-pressure properties of diet are not limited to these specific foods and/or nutrients. In this regard, the systematic literature review and meta-analysis by Aboud investigated whether vitamin D supplementation reduces BP in children and adolescents [6]. Although this review included both non-randomized and randomized controlled clinical trials, it did not report any significant effect of vitamin D supplementation in lowering SBP and DBP in children and adolescents. While there is evidence indicating a modest BP-lowering effect of vitamin D in adult patients who are hypertensive, the effect of vitamin D on the blood pressure levels of normotensive adults has not been found to be significant [7,8]. Furthermore, the review by Tsirimiagkou et al. gathered existent clinical trials investigating the effect of sodium intake or sodium reduction in subclinical vascular damage like atherosclerosis, atheromatosis and arterial remodeling. It was evidenced that most of the relevant interventions mainly investigate arteriosclerosis, showing either reduction of arterial stiffness through sodium intake reduction or no statistically significant effects. Regarding arterial remodeling and atheromatosis, it was highlighted that there is scarce evidence, which does not allow solid conclusions. Lastly, the Special Issue also included another review by Malinowski et al. that examined the available evidence on the effect of a range of dietary compounds on blood pressure levels [9]. This review reported that there is good evidence suggesting the blood pressure-lowering effect of lycopene, docosahexaenoic acid (i.e., omega-3 fatty acid), epigallocatechin-3-gallate (i.e., a type of catechin found in tea) and dietary fibre, although further studies are required to strengthen these findings.

In conclusion, despite the wide range of pharmacological treatments for high blood pressure control, hypertension remains one of the leading health problems, highlighting the need for additional, non-pharmacological solutions. Diet therapy has been repeatedly proven to be effective in blood pressure control, either as a whole through healthy dietary patterns, such as DASH or the Mediterranean diet, or through bioactive nutrients that include vasoactive compounds. Future experimental studies should aim to assess the long-term compliance to these healthy dietary patterns, as well as the effective and safe dose of individual dietary compounds and their combination in the control of high blood pressure.
